# Near-field imaging of buried conductors using transient EM signal transfer

**DOI:** 10.1038/s41598-026-46396-y

**Published:** 2026-04-03

**Authors:** Tomáš Doležal, Martin Štumpf

**Affiliations:** 1 Department of Radio Electronics, Faculty of Electrical Engineering and Communication, Brno University of Technology, Technicka 12, 616 00 Brno, Czech Republic; 2 Department of Computer Science, Electrical and Space Engineering, EISLAB, Luleå University of Technology, 971 87 Luleå, Sweden

**Keywords:** EM imaging, EM coupling, Time-domain analysis, Non-invasive approach, Subsurface detection, Buried conductors, Engineering, Physics

## Abstract

A novel time-domain electromagnetic (TDEM) technique for reliable detection and imaging of visually obscured buried conductors using only two rapidly acquired measurements via a simple and cost-effective antenna system is presented. This non-invasive method exploits transient electromagnetic (EM) interactions to detect contrasts in electrical conductivity between targets and the surrounding medium. This approach provides a practical and flexible alternative to conventional subsurface sensing technologies. The presented results show that the proposed technique yields accurate and repeatable outcomes across various shapes of buried targets.

## Introduction

Accurate detection and characterization of subsurface inhomogeneities–such as hidden unexploded ordnance (UXO), structural defects, and pathological tissue alterations–are essential in diverse fields. These include geophysical prospecting^1,2^, military defense^3,4^, structural health monitoring^5,6^, and biomedical diagnostics^7,8^. Indeed, the reliable identification of such inhomogeneities is crucial to ensure safety, maintain structural integrity, enable early diagnosis, and mitigate security risks.

Variations in signal acquisition hardware and processing algorithms fundamentally determine the strengths and limitations of EM sensing techniques. Traditional frequency-domain EM (FDEM) methods analyze steady-state or frequency-specific responses to extract material EM properties. However, various techniques, such as ground penetration radar^9,10^ (GPR) and eddy current testing^11,12^ (ECT), incorporate time-domain (TD) processing to capture transient signals. This hybrid approach facilitates better target resolution and enhanced noise immunity by harnessing temporal decay characteristics, resonance phenomena, and dispersive behaviors that are not readily accessible in pure FDEM analysis. Nonetheless, these dual-domain methods pose challenges in hardware, data acquisition, and computation.

Over the past decades, time-domain EM (TDEM) methods have emerged as a particularly promising approach to near-surface investigation. As detailed in Nabighian’s seminal work, TDEM techniques may exploit the analysis of transient secondary fields to enable sensitive detection of conductive targets compared to FDEM techniques^13^. Chen and Peters show that complex natural resonances (CNRs) in TD backscattered EM signals enable a robust, orientation-invariant subsurface target characterization^14^. Recent studies show that TDEM methods, combined with resistivity and seismic imaging, excel at delineating water-rich zones, altered rock boundaries, and underground voids, especially in geologically complex mining settings where conventional FDEM approaches face limitations^15,16^.

In the context of UXO, traditional FDEM methods such as magnetometry, EM induction (EMI), and GPR frequently produce numerous false positives. To improve target detection, singularity expansion methods (SEM) exploit the natural resonant frequencies of the objects by analyzing their EM, magnetic, and acoustic responses. However, these advanced approaches often involve high costs and significant computational effort, which limits their widespread use^17^.

In this paper, we present a fully TDEM method that achieves reliable detection and image of visually obscured targets using only two rapidly acquired measurements via a simple and cost-effective antenna system. This approach balances operational efficiency and accuracy by relying solely on contrast between the target and its surroundings, while allowing scalability for improved resolution through additional measurements. The method extends a non-invasive EM technique, originally developed for measuring human tissue conductivity^18^ as based on the analytical framework^19,20^, to the detection and identification of metallic objects. It exploits the interaction of transient EM fields with buried metallic targets, enabling their identification through the close-range coupling between two loop antennas positioned above the ground.

## Problem definition

**Fig. 1:**
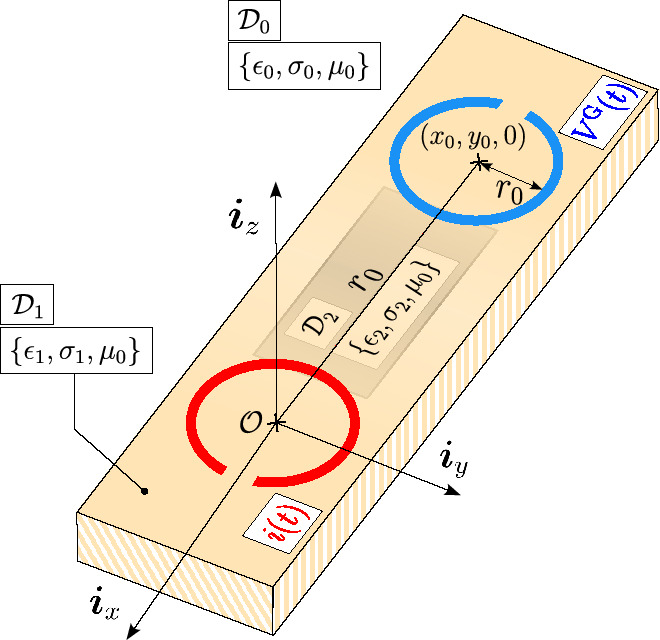
Transmitting and receiving loop antennas situated on the examined material sample $\mathcal {D}_{1}$ in presence of a buried conductor occupying a bounded domain $\mathcal {D}_2$.

The starting point of the analysis is the problem configuration depicted in Fig. 1. Position in the examined 3-D space $\mathbb {R}^3$ is specified by coordinates $\{x, y, z\}$ with respect to the Cartesian coordinate system with the origin $\mathcal {O}$ and the three mutually perpendicular unit vectors $\{ \boldsymbol{\textit{i}}_x, \boldsymbol{\textit{i}}_y, \boldsymbol{\textit{i}}_z \}$ forming the standard base.

The problem configuration consists of two thin-wire loop antennas located in the plane separating two dissipative half-spaces $\mathcal {D}_{0,1}$. The EM constitutive properties of $\mathcal {D}_{0,1}$ are described by their electric permittivity $\epsilon _{0,1}$, electric conductivity $\sigma _{0,1}$, and magnetic permeability $\mu _{0}$, with corresponding EM wave speeds $c_{0,1} = (\epsilon _{0,1} \mu _{0,1})^{-1/2}$. Within $\mathcal {D}_1$, a metallic object $\mathcal {D}_2$ is buried, characterized by its own EM properties: $\{ \epsilon _2, \sigma _2, \mu _0 \}$. In practical scenarios, the lower half-space, $\mathcal {D}_1$, typically represents the ground, while the upper one, $\mathcal {D}_0$, is free space. The transmitting loop is assumed to be located at the origin, while the receiving antenna is placed at a distance $r_0 = (x_0^2 + y_0^2)^{1/2}$ far apart. The respective areas of the transmitting and receiving loops are denoted by $\mathcal {A}_T$ $=$ $\mathcal {A}_R$ $=$ $\pi r^2$. The transmitting loop is activated by an excitation electric current pulse, denoted as $i(t)$. Consequently, the voltage response $V^\textrm{G}(t)$ is induced across the ports of the receiving loop. The voltage signal is to be measured and further processed using the algorithm closely described in^18^ or summarized below in. In this experimental scenario, $\mathcal {D}_2$ represents a metallic object buried within the region $\mathcal {D}_1$. The unbounded region $\mathcal {D}_0$ surrounding $\mathcal {D}_1$ is assumed to be free space.

## Methodology

In this section, we describe the applied imaging methodology including the pertaining signal processing technique. The measurement setup used for this practical experiment is the same as in^18^. The methodology is divided into four main parts. First, the algorithm^18^ used for the extraction of the effective electrical conductivity is summarized. Next, the fundamental principle of the measurement approach is introduced, using two antennas to illustrate the underlying concept. Further, a specific antenna field configuration is presented, along with its associated scanning procedure to examine the target area. In the last section, the signal processing techniques applied to the measured data are discussed.

### Algorithm for electrical conductivity inversion

The core principle lies in comparing the measured voltage response $V_\text {M}^\text {G}(t)$ with a theoretically calculated response $V_\text {T}^\text {G}(t)$. After acquiring the measured voltage response $V_\text {M}^\text {G}(t)$, the signal is imported into the algorithm as input for subsequent processing. The main function of the algorithm is to calculate the theoretical voltage response $V_T^{G}(t)$ for specific predefined conductivity values $\sigma _T \in [\sigma _A, \sigma _B]$, using the following equation, derived in^20^.

1 $$\begin{aligned} \lim _{\sigma _{0}\downarrow 0}V_\textrm{T}^{\textrm{G}}(t, \sigma _\textrm{T}(j))\simeq&\big (\mathcal {A}^{\textrm{T}}\mathcal {A}^{\textrm{R}}\big /2\pi r_{0}^{5}\big )\sigma _\textrm{T}(j)^{-1}~ \hspace{0pc}\partial _{t}i(t) {*}_{{}_{t}}{\,}[9\,\textrm{erf}(T/2t^{1/2}) \!- \!T(\pi t)^{-1/2}(9 \!+ \!3T^{2}/2t\! + \!T^{4}/4t^{2}) \nonumber \\&\hspace{-7.8pc}\times \,\exp (-T^{2}/4t)]\textrm{H}(t). \end{aligned}$$

where $T = r_0(\sigma _1 \mu _0)^{1/2}$, $\textrm{erf}(x)$ denotes the error function and index $j \in [1, P]$,where *P* is the number of division points, determines the specific value of $\sigma _\textrm{T}$ in the input sequence $[\sigma _\textrm{A}, \sigma _\textrm{B}]$. The time coordinate is denoted by *t*, time convolution is $*_t$ and time-derivative operator is $\partial _t$. To quantify the difference between the theoretically computed voltage response samples $V_\textrm{T}^{\text {G}}(n \Delta t, \sigma _\textrm{T}(j))$ and the actual measured voltage response, we used the root-mean-square error ($\textrm{RMSE}$) evaluation as follows

2 $$\begin{aligned} \textrm{RMSE}(\sigma _\textrm{T}(j)) = \sqrt{\frac{1}{N}\sum _{n=1}^N [V_\textrm{T}^{\text {G}}(n \Delta t, \sigma _\textrm{T}(j)) - V_\textrm{M}^{\text {G}}(n \Delta t)]^2} \end{aligned}$$

where $\Delta t> 0$ is the time step. The time step is chosen to be a small fraction of the pulse time width, $t_\textrm{w}$, and the length of the time window of observation is taken to be its multiple, e.g., $T_\textrm{tot} = 3t_\textrm{w}$, which is sufficient to capture the diffusive propagation effects. Afterwards, the electrical conductivity $\sigma$ can be determined by detecting the minimum value of the $\textrm{RMSE}$ function

3 $$\begin{aligned} \sigma _1 = \textrm{argmin}_{\sigma _\textrm{T} \in [\sigma _\textrm{A}, \sigma _\textrm{B}]} \textrm{RMSE}(\sigma _\textrm{T}(j)) \end{aligned}$$

### Measurement principle

The employed analytical model describes the transient EM signal transfer between two loop antennas located on the ground. The model assumes that the ground is homogeneous in its EM properties. However, in this experimental scenario, the presence of a buried metallic object introduces a significant EM inhomogeneity, thereby violating this underlying assumption. Despite this, it remains suitable for this study, as the primary objective is not the precise estimation of absolute electrical conductivity, but the identification of EM inhomogeneities within the subsurface. The inherent limitation of the method, in which the transmitting and receiving loops interpret the medium as homogenized, results in a spatially averaged conductivity value. This value does not reflect the true conductivity of the embedded metallic object, but rather the contrast it introduces relative to the surrounding medium. Therefore, it is appropriate to introduce the term effective conductivity $\sigma _\textrm{e}$, a derived quantity that reflects the presence and spatial distribution of the object, rather than its intrinsic electrical properties.

**Fig. 2:**
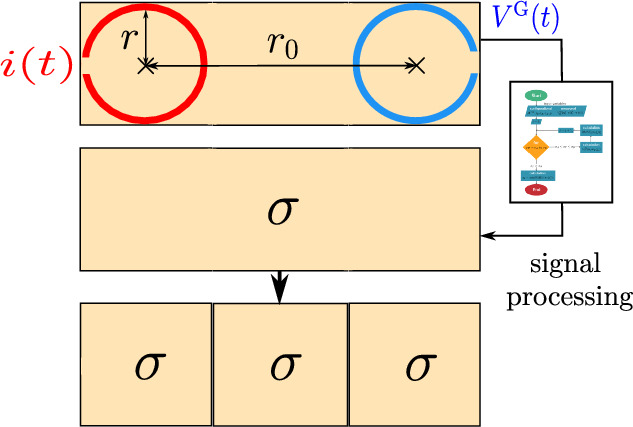
Illustration of constructing the conductivity vector from single measurement.

The area between the transmission and the receiving loops is divided into uniform cells, each assigned the same $\sigma _\textrm{e}$ value obtained from a single measurement. Essentially, the measured conductivity is considered as an average conductivity for the entire area beneath the loops in a single measurement. This results in the creation of a conductivity vector for the scanned region beneath the loops, as illustrated in Fig. 2, which is then used as input for the signal processing block. The process of creating the conductivity vector is shown in Fig. 2

The methodology for extracting $\sigma _\textrm{e}$ is based on the approach described in^18^. The signal processing block in Fig. 2 implements the same algorithm used in^18^ to calculate the conductivity of human tissue.

**Fig. 3:**
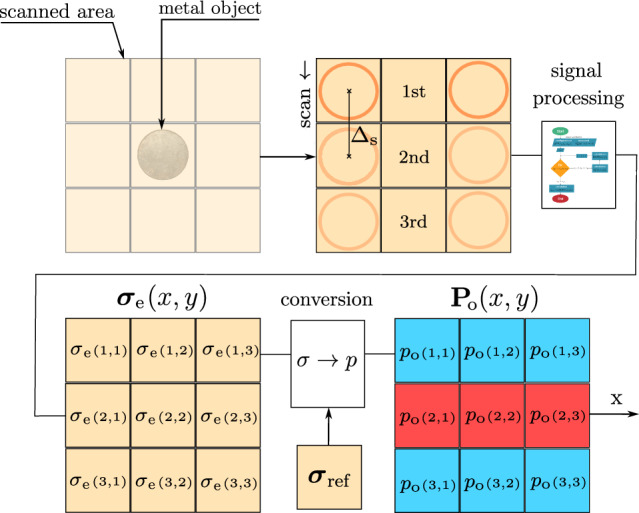
Block diagram representing the process of constructing the probability matrix of a scanned 2D field.

To calibrate the system before the main measurement, the electrical conductivity of ground must be determined in the absence of any object beneath the loop antennas, providing a reference value $\sigma _{\textrm{ref}}$. This reference conductivity is then compared with the conductivity $\sigma _{\textrm{e}}$ obtained in the presence of a contrasting conductive object. The resulting deviation, calculated in the conversion block shown in Fig. 3, is used to estimate the probability of the object being located at a specific position within the 2D field under examination as

4 $$\begin{aligned} \textbf{P}_{\text {o}}(x, y) = \frac{\left| \boldsymbol{\sigma }_{\text {e}}(x, y) - \boldsymbol{\sigma }_{\text {ref}}(x, y) \right| }{\max \left( \left| \boldsymbol{\sigma }_{\text {e}}(x, y) - \boldsymbol{\sigma }_{\text {ref}}(x, y) \right| \right) } \end{aligned}$$

where, $x$ and $y$ represent the coordinates in the 2D field, $\textbf{P}_{\text {o}}(x, y)$ denotes the matrix of probabilities of the presence of object at $(x, y)$, $\boldsymbol{\sigma }_{\text {e}}(x, y)$ is the matrix of measured effective electrical conductivities at $(x, y)$ with the object, and $\boldsymbol{\sigma }_{\text {ref}}(x, y)$ is the matrix of reference conductivities at $(x, y)$ without the object. In this work, the term probability refers to a normalized quantity that represents the relative change of the measured parameter with respect to the background. It is not a statistical probability, but a percentage-based measure of relative contrast.

Subsequently, the thus computed probability values are used as inputs for the signal processing algorithm (see Section Signal Processing for Data Analysis), to determine the location and shape of the object. The measurement principle is illustrated in Fig. 3, demonstrating the detection of a metal object positioned at the center of the examined 2D field. The probability matrix $\textbf{P}_{\text {o}}(x, y)$ is obtained from three individual measurements, each performed with a step size $\Delta _\textrm{S} = 2r$ using a pair of loops.

### Antenna configuration and scanning method

In this section, we describe the antenna array configuration as used in the experiment, along with the scanning methodology applied to the examined area. The antenna array has been designed and manufactured to estimate the shape and position of the object with the minimal number of measurements while also optimizing the number of antennas used. Although different antenna array configurations could be employed, the fundamental principle would remain unchanged.

**Fig. 4:**
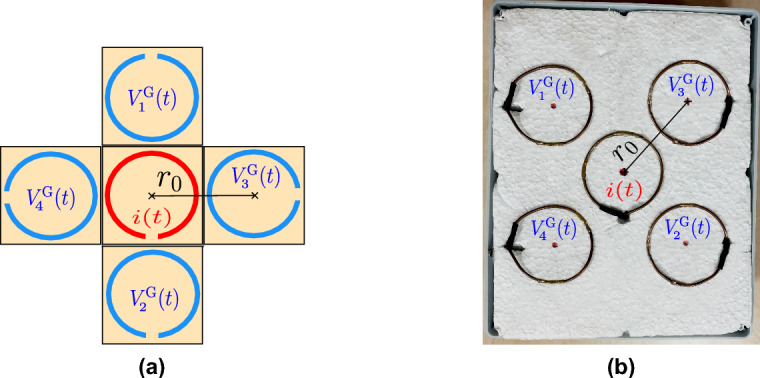
Configuration of the antenna array with a central transmitting loop (red) and four receiving loops (blue): (**a**) theoretical schematic of the antenna array (**b**) practical implementation used in this study.

The antenna array consists of five loop antennas, one transmitting and four receiving (see Fig. 4). All antennas are placed in the same horizontal plane, with the central antenna acting as the transmitter that is surrounded by four receiving probes.

The scanning methodology employs the specified antenna array to acquire transient EM data. Conceptually, a $3 \times 3$ grid can be considered beneath the antenna array, where each transmitter-receiver pair provides conductivity values and the probability of presence of an object. The scanning process is conducted in two steps, which can be seen as a stamping method, effectively capturing two “stamps” of data.

**Fig. 5:**
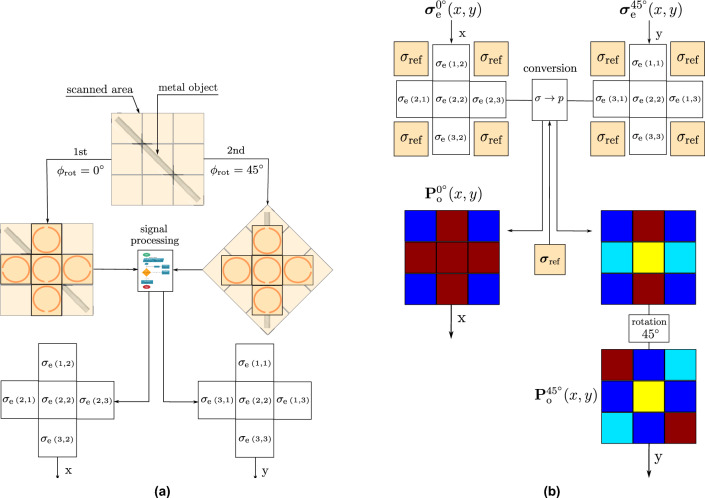
Measurement and conversion of electrical conductivity distributions for two antenna array orientations. (**a**) Measurement of electrical conductivity matrix $\boldsymbol{\sigma }_{\text {o}}(x, y)$ for $\phi _\textrm{rot} = \{0^\circ ; 45^\circ \}$ in a scanned area with a buried metal object using a 5-loop antenna array configuration. (**b**) The conversion process of the electrical conductivity matrix $\boldsymbol{\sigma }_{\text {e}}(x, y)$ for $\phi _\textrm{rot} = \{0^\circ ; 45^\circ \}$ into the corresponding probability maps $\textbf{P}_{\text {o}}^{0^\circ }(x, y)$ and $\textbf{P}_{\text {o}}^{45^\circ }(x, y)$.

In the first step, the electrical conductivity values are measured with each transmitter-receiver pair of loops for the first configuration that corresponds to $\phi _\textrm{rot} = 0^\circ$ as shown in Fig. 5a. These values correspond to ${\sigma }_{\text {e}}(1, 2)$, ${\sigma }_{\text {e}}(2, 1)$, ${\sigma }_{\text {e}}(2, 3)$, and ${\sigma }_{\text {e}}(3, 2)$ in the 3x3 conductivity matrix $\boldsymbol{\sigma }_{\text {e}}(x, y)$. The value at position ${\sigma }_{\text {e}}(2, 2)$ is calculated as the average of the surrounding values, while other positions are substituted with the reference soil conductivity ${\sigma }_{\text {ref}}$, as depicted in Fig. 5b. This generates a probability map $\textbf{P}_{\text {o}}^{0^\circ }(x, y)$ for $\phi _\textrm{rot} = 0^\circ$ via (4).

In the second step, the same procedure is repeated for the antenna array rotated by $\phi _\textrm{rot} = 45^\circ$ (see Fig. 5a). This yields the second probability map $\textbf{P}_{\text {o}}^{45^\circ }(x, y)$ shown in Fig. 5b. Lastly, the probability map obtained using the $45^\circ$-rotated antenna array but represented in the $0^\circ$ reference frame, is rotated by $45^\circ$ to restore the correct spatial positions. Both maps are then used as inputs in the signal processing block (see Section Signal Processing for Data Analysis).

In both steps, the antenna array provides valid measurements only at the central and edge positions. The four corner entries of the 3 $\times$ 3 data matrix are not measured and are temporarily assigned the reference conductivity $\sigma _{\textrm{ref}}$ solely to preserve matrix dimensionality for rotation and signal processing. These placeholder values are overwritten during the data-combination step and therefore have no influence on the reconstructed image.

### Signal processing for data analysis

**Fig. 6:**
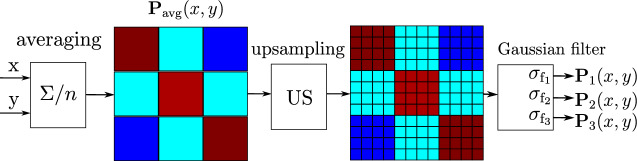
Averaging of probability maps $P_{\text {o}}^{0^\circ }(x, y)$ and $P_{\text {o}}^{45^\circ }(x, y)$, followed by upsampling and smoothing with Gaussian filter to produce the final probability maps.

This section outlines the processing of two probability maps generated for $\phi _\textrm{rot} = 0^\circ$ and $\phi _\textrm{rot} = 45^\circ$ antenna field orientations. This procedure is illustrated in Fig. 6. In the initial stage of processing, these maps are combined via point-wise averaging to produce a unified and normalized probability map as

5 $$\begin{aligned} \textbf{P}_{\text {avg}}(x, y) = \frac{ \frac{1}{2} \left( \textbf{P}_{\text {o}}^{0^\circ }(x, y) + \textbf{P}_{\text {o}}^{45^\circ }(x, y) \right) }{ \displaystyle \max _{x, y} \left[ \frac{1}{2} \left( \textbf{P}_{\text {o}}^{0^\circ }(x, y) + \textbf{P}_{\text {o}}^{45^\circ }(x, y) \right) \right] } \end{aligned}$$

This probabilistic map originates from a 3 $\times$ 3 coarse measurement grid, which produces sharp cell-to-cell transitions that reflect the sampling layout rather than the true spatial behavior of the field. In order to obtain a continuous and spatially coherent conductivity distribution, the coarse map is first upsampled so that each original measurement cell is represented by a dense block of pixels (see Fig. 6). A Gaussian filter is then applied to suppress the artificial block boundaries and to introduce smooth spatial gradients across the map, providing a suitable input for the subsequent processing steps.

Consequently, three probabilistic maps are generated for each scan area by smoothing the averaged and upsampled map with Gaussian filters of standard deviations $\sigma _{\textrm{f}_1}, \sigma _{\textrm{f}_2}, \sigma _{\textrm{f}_3}$, yielding the final probability maps $\textbf{P}_{1}(x, y), \textbf{P}_{2}(x, y), \textbf{P}_{3}(x, y)$.

These maps are further processed using two independent techniques: binary segmentation via thresholding and morphological transformation, each aiming to enhance object localization and shape estimation.

#### Binary segmentation via thresholding

The thresholding operation is applied to the probabilistic map $\textbf{P}_{n}(x, y)$ to obtain a binary map $\textbf{B}_{n}(x, y)$, where *n* denotes the index of the given probabilistic map, as defined by:

6 $$\begin{aligned} \textbf{B}_{n}(x, y) = {\left\{ \begin{array}{ll} 1, & \text {if } \textbf{P}_{n}(x, y) \ge T_{\textrm{th}} \\ 0, & \text {if } \textbf{P}_{n}(x, y) < T_{\textrm{th}} \end{array}\right. } \end{aligned}$$

where $T_{\textrm{th}}$ is the threshold value. This process converts the probability values into a binary representation, indicating the presence (1) or absence (0) of the object at the specific location (*x*, *y*). Thresholding enables the extraction of the object shape and its localization by isolating the most probable regions within the map.

#### Binary image refinement via morphology

In this procedure, dilation, erosion, and gradient operations were applied to probabilistic maps with continuous values to refine object boundaries and improve detection accuracy. These operations use a structural element $S$. This element $S$ defines the local neighborhood used in morphological operations and is represented as a discrete disk

7 $$\begin{aligned} S = \left\{ (x', y') \in \mathbb {Z}^2 \;\Bigg |\; \sqrt{x'^2 + y'^2} \le r_{S} \right\} \end{aligned}$$

where $x'$ and $y'$ are spatial offsets relative to the center. The radius $r_{S}$ determines the extent of the neighborhood applied around each pixel.

- **Dilation** ($\oplus$) expands high-probability regions by assigning each pixel the maximum value within its $S$-defined neighborhood:
  8 $$\begin{aligned} \textbf{P}_{\oplus }(x, y) = \max _{(x', y') \in S} \textbf{P}(x + x', y + y') \end{aligned}$$
- **Erosion** ($\ominus$) shrinks these regions by assigning each pixel the minimum value within its neighborhood:
  9 $$\begin{aligned} \textbf{P}_{\ominus }(x, y) = \min _{(x', y') \in S} \textbf{P}(x + x', y + y') \end{aligned}$$
- The **morphological gradient** ($\textbf{P}_{\text {grad}}$) highlights object boundaries by calculating the difference between the dilated and eroded maps:
  10 $$\begin{aligned} \textbf{P}_{\text {grad}}(x, y) = \textbf{P}_{\oplus }(x, y) - \textbf{P}_{\ominus }(x, y) \end{aligned}$$

These operations improve object localization and reduce noise in probabilistic maps. The shape of $S$ and size control the spatial extent of the operation on each pixel, allowing for refined object shapes and enhanced detection accuracy.

## Results

In this section, we present the results that have been achieved through the methodology discussed in section Methodology. The primary objective of this experiment is to validate the applicability of the introduced methodology for the localization and shape estimation of buried metallic objects. First, we provide an overview of the measurement setup, including a description of the manufactured antenna array employed in the experiment. In addition, metallic test objects are introduced and characterized. Finally, the results obtained with both processing techniques are presented.

To evaluate the location and shape of an object, the algorithm originally developed to estimate the electrical conductivity of biological tissues^18^ was employed to calculate apparent conductivity contrasts within the scan area. The conductivity values were then calculated from the measurements acquired with and without the buried metallic object, and the resulting differences were used to generate probability maps. These maps represent the estimated location of the object, based on changes in the EM response caused by conductivity differences in the surrounded medium.

### Measurement setup and excitation pulse

**Fig. 7:**
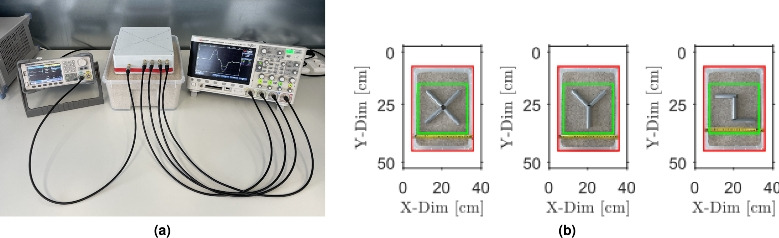
Experimental setup and object configurations used in measurement procedure. (**a**) The configuration of the actual measurement workplace. (**b**) Configurations of the objects used for experiment.

The experimental setup (see Fig. 7a) consists of a custom-designed loop antenna array, an arbitrary waveform generator Siglent SDG6022X, and a 4-channel digital oscilloscope Keysight DSOX2024A. The practical implementation of the antenna array employed in this study is illustrated in Fig. 4b. The metallic object was completely buried beneath a $5\,\textrm{mm}$ layer of silica sand inside a $37 \times 26 \times 12\,\textrm{cm}$ plastic box outlined by the red boundary in Fig. 7b. The silica sand provided a controlled and homogeneous surrounding medium for subsurface imaging.

Three metallic configurations of the object were composed of hollow circular steel sections with a diameter of 20 mm to evaluate the ability of the method to detect objects of various geometric complexity. The object shapes are represented with letters X, Y and Z, as shown in Fig. 7b. The green boundary delineates the $20 \times 20\,\textrm{cm}$ scanning region.

The antenna array consists of five loop antennas of radius of $r = 30\,\textrm{mm}$ arranged in a fixed geometry with the central element acting as the transmitter and the remaining four as receivers (see Fig. 4). The antennas are evenly spaced with a distance $r_0 = 7.5 \,\textrm{cm}$ between the transmitting and each receiving loop. They are enclosed within a plastic box with all outputs routed externally via SMA connectors. Each antenna was constructed from a $2\, \textrm{mm}$ thick copper wire to provide sufficient mechanical stability.

**Fig. 8:**
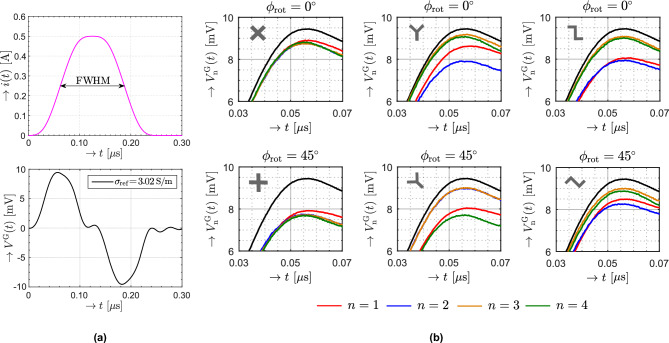
Measured voltage responses under controlled conditions. (**a**) The response recorded in the absence of a buried object. (**b**) Measured responses with amplitude variation due to presence of examined objects, shown for both antenna array orientation $\phi _{\textrm{rot}} = 0^\circ$, $\phi _{\textrm{rot}} = 45^\circ$.

The transmitting loop was excited by a continuously differentiable, time-windowed current pulse as defined in reference^19^ and shown in Fig. 8a. However, the methodology is not restricted to this specific waveform. Under the assumption that the receiving loop is positioned sufficiently close to the transmitting loop, the contribution of displacement currents can be neglected, and the induced open-circuit voltage can be attributed to conduction currents^20^. This quasi-static approximation is valid when the condition $r_0 \ll c_0 t_\textrm{w}$ is satisfied, where $t_\textrm{w}$ corresponds to the full width at half maximum duration (FWHM) illustrated in Fig. 8a. In accordance with this requirement, we set $t_\textrm{w} = 125\,\textrm{ns}$. The amplitude of the exciting electric-current pulse was set to $i_\textrm{m} = 0.5\,\textrm{A}$. It ensures that the induced voltage at the receiving loop remained measurable with the available instrumentation. This parameter serves solely to achieve an adequate signal level and does not affect the underlying physical model.

### Measured data and analysis

In the initial step of the experiment, the voltage response was recorded in the absence of any object to determine the reference effective conductivity $\sigma _{\textrm{ref}}$. Effective conductivity reflects the presence of EM inhomogeneities within the subsurface region beneath the loops rather than intrinsic conductivity. The corresponding measured response $V^{\text {G}}(t)$, accompanied by the value of $\sigma _{\textrm{ref}}$, is presented in Fig. 8a.

Afterward, this process was repeated for all three metallic object configurations. The responses $V^{\text {G}}_{n}(t)$ with $n=1,2,3,4$ denoting the receiving antenna index, were acquired from all receiving antennas in both antenna array orientations $\phi _{\textrm{rot}}$. Figure 8b provides a comprehensive representation of the measured responses. To highlight the amplitude differences caused by the presence of an object, a reduced time window was selected for visualization. The black curve shows the reference response measured without any object from Fig. 8a.

The calculated components of the effective conductivity matrices $\boldsymbol{\sigma }_{\textrm{e}}(x, y)$ corresponding to the antenna array orientation $\phi _{\textrm{rot}} = 0^\circ$, $\phi _{\textrm{rot}} = 45^\circ$ and all examined objects are presented as follows

$$\begin{aligned} & \begin{array}{@{\hspace{2cm}} c@{\hspace{2cm}}c} \boldsymbol{\sigma }_{\text {e}, \text {X}}^{0^\circ }(x, y) = \begin{bmatrix} 3.02 & 2.68 & 3.02 \\ 2.46 & 2.49 & 2.42 \\ 3.02 & 2.40 & 3.02 \end{bmatrix} & \boldsymbol{\sigma }_{\text {e}, \text {X}}^{45^\circ }(x, y) = \begin{bmatrix} 3.02 & 1.56 & 3.02 \\ 1.38 & 1.45 & 1.41 \\ 3.02 & 1.43 & 3.02 \end{bmatrix} \end{array} \\ & \begin{array}{@{\hspace{2cm}}c@{\hspace{2cm}}c} \boldsymbol{\sigma }_{\text {e}, \text {Y}}^{0^\circ }(x, y) = \begin{bmatrix} 3.02 & 2.20 & 3.02 \\ 2.94 & 2.41 & 2.96 \\ 3.02 & 1.54 & 3.02 \end{bmatrix} & \boldsymbol{\sigma }_{\text {e}, \text {Y}}^{45^\circ }(x, y) = \begin{bmatrix} 3.02 & 1.62 & 3.02 \\ 1.40 & 2.20 & 2.90 \\ 3.02 & 2.89 & 3.02 \end{bmatrix} \end{array} \\ & \begin{array}{@{\hspace{2cm}}c@{\hspace{2cm}}c} \boldsymbol{\sigma }_{\text {e}, \text {Z}}^{0^\circ }(x, y) = \begin{bmatrix} 3.02 & 1.65 & 3.02 \\ 2.77 & 2.21 & 2.79 \\ 3.02 & 1.62 & 3.02 \end{bmatrix} & \boldsymbol{\sigma }_{\text {e}, \text {Z}}^{45^\circ }(x, y) = \begin{bmatrix} 3.02 & 1.71 & 3.02 \\ 2.47 & 2.10 & 2.44 \\ 3.02 & 1.78 & 3.02 \end{bmatrix} \end{array} \end{aligned}$$

**Fig. 9:**
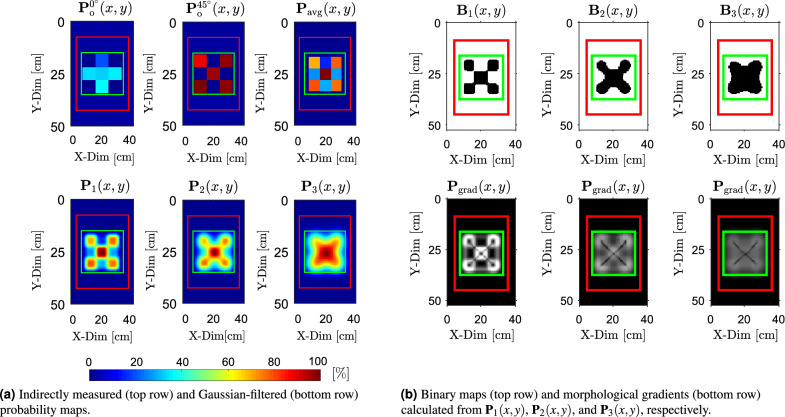
Results of detection for object “X” buried beneath a 5 mm layer of silica sand. (**a**) Raw and filtered probability maps, while (**b**) presents the corresponding binary segmentations and morphological gradients calculated from the probability maps.

**Fig. 10:**
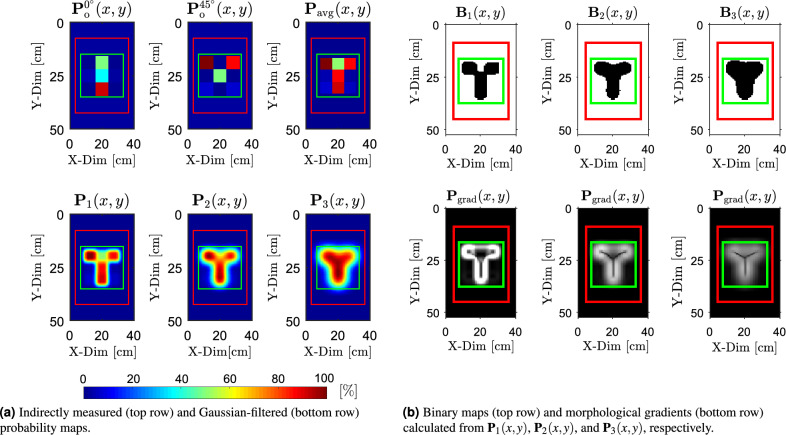
Results of detection for object “Y” buried beneath a 5 mm layer of silica sand. (**a**) Raw and filtered probability maps, while (**b**) presents the corresponding binary segmentations and morphological gradients calculated from the probability maps.

**Fig. 11:**
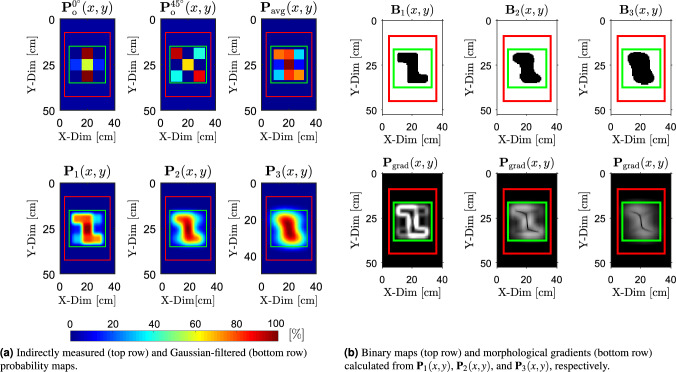
Results of detection for object “Z” buried beneath a 5 mm layer of silica sand. (**a**) Raw and filtered probability maps, while (**b**) presents the corresponding binary segmentations and morphological gradients calculated from the probability maps.

Furthermore, the effective conductivity matrices $\boldsymbol{\sigma }_{\text {e}, \text {X}}(x, y)$, $\boldsymbol{\sigma }_{\text {e}, \text {Y}}(x, y)$, and $\boldsymbol{\sigma }_{\text {e}, \text {Z}}(x, y)$ were converted into the corresponding probability maps $P_{\text {o}}^{0^\circ }(x, y)$ and $P_{\text {o}}^{45^\circ }(x, y)$, following the procedure illustrated in Fig. 5b. Consequently, these maps were used to calculate the averaged probability map $P_{\text {avg}}(x, y)$, as defined in (5). These maps are presented in the first row of Figs. 9a, 10a, and 11a for each examined object.

Next, the Gaussian filtering was applied to smooth spatial variations in the probability distributions and to compensate for the assumption of locally uniform conductivity beneath each pair of coils as shown in Fig. 2. The resulting smoothed maps, denoted as $\textbf{P}_1(x, y)$, $\textbf{P}_2(x, y)$, and $\textbf{P}_3(x, y)$, were calculated with standard deviations $\sigma _{\textrm{f}_1} = 2$, $\sigma _{\textrm{f}_2} = 4$, and $\sigma _{\textrm{f}_3} = 6$, respectively. These filtered maps are shown in the second row of Figs. 9a, 10a, and 11a. These standard deviations were chosen through a structured empirical evaluation to provide progressively stronger spatial smoothing. Increasing $\sigma _{\textrm{f}}$ produces smoother probability maps and gradually suppresses the edges of artificial blocks introduced by coarse 3 $\times$ 3 sampling, while still preserving the spatial extent of the buried object. Across all results, this empirical approach yielded stable and interpretable probability maps and provides a reliable basis for the binarization and morphological operations.

The binarized versions of $\textbf{P}_1(x, y)$, $\textbf{P}_2(x, y)$, and $\textbf{P}_3(x, y)$ are shown in the first rows of Figs. 9b, 10b, and 11b. A fixed threshold of $T_{\textrm{th}} = 50 \%$ was applied across all objects and Gaussian filter settings to obtain the corresponding binary maps $\textbf{B}_1(x, y)$, $\textbf{B}_2(x, y)$, and $\textbf{B}_3(x, y)$. A threshold of $T_{\textrm{th}} = 50 \%$ was selected because it represents a neutral, scale-independent midpoint of the normalized probabilistic map. This ensures a robust and unbiased separation between target responses and background. The areas influenced by the target generally exceed this level, whereas the background remains below it across all smoothing configurations.

Morphological gradients $\textbf{P}_\text {grad}(x, y)$ calculated from $\textbf{P}_1(x, y)$, $\textbf{P}_2(x, y)$, and $\textbf{P}_3(x, y)$ are shown in the second rows of Figs. 9b, 10b, and 11b, using disk-shaped structuring elements with radii $r_S = 3$, 4, and 6, respectively. The choice of the structuring-element radii $r_S$ follows directly from the physical scale of the experiment. Each hollow circular steel section used to create the objects (see Fig. 7b) has a diameter of 20 mm. The scanning area (20 $\times$ 20 cm), marked by the green boundary in Fig. 7b, is sampled by a 3 $\times$ 3 grid, producing an initial cell size of $\approx 66.7$ mm. After upsampling $\textbf{P}_{\text {avg}}(x, y)$ by a factor $u = 40$, each cell is represented by a 40 $\times$ 40 pixel block, resulting in an effective pixel size of approximately $\approx 1.67$ mm (see Fig. 6). In this representation, the 20 mm tube width spans about 12 pixels. To avoid boundary deformation during morphological operations, the structuring-element radius $r_S$ must therefore remain below or equal to half of this width. The selected radii of 3–6 pixels (corresponding to $\approx 5-10$ mm) satisfy this requirement. The finer probabilistic maps (lower $\sigma _\textrm{f}$) use the smaller radius, while smoother maps (higher $\sigma _\textrm{f}$) allow for a slightly larger radius because their boundaries vary more gradually and remain stable.

### Reconstruction accuracy analysis

The reconstructed probability maps (Figs. 9a, 10a, and 11a) are calculated from the spatially averaged value of $\sigma _{\textrm{e}}$, obtained from the antenna array measurements. The spatial averaging corresponds to the sensing area of the loop antennas (see Fig. 2). As a result, the reconstructed object exhibits a spatially extended representation relative to its true geometry. In this context, the normalized mean absolute error ($\textrm{NMAE}$) and the skeleton-based intersection-over-union ($\textrm{IoU}_{\textrm{skel}}$) were used as complementary evaluation metrics to characterize different aspects of reconstruction quality. All evaluations were performed within a predefined region of interest (ROI), indicated by the green rectangle in Figs. 9, 10, and 11.

**Fig. 12:**
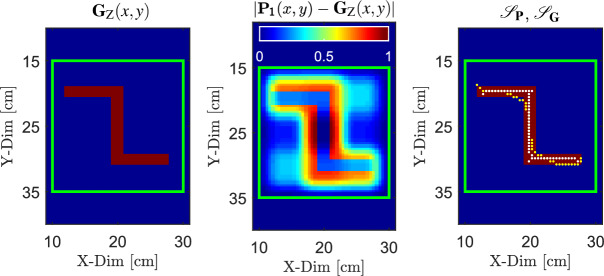
Evaluation of reconstruction accuracy for object “Z”. The left panel shows the reference mask $\textbf{G}_\textrm{Z}(x,y)$ derived from the true object geometry shown in Fig. 7b. The middle panel visualizes the absolute difference $\left| \textbf{P}_1(x,y) - \textbf{G}_\textrm{Z}(x,y) \right|$ within the ROI used for NMAE computation, corresponding to the probability map $\textbf{P}_1(x,y)$ shown in Fig. 11a. The right panel shows the skeleton-based geometric comparison, where one-pixel-wide skeletons of the reference mask (white dotted line) and the reconstructed probability map (yellow dotted line) are used to calculate IoU$_{\textrm{skel}}$.

The $\textrm{NMAE}$ measures the average pixel-wise deviation between the probability maps $\textbf{P}_1(x,y)$, $\textbf{P}_2(x,y)$, and $\textbf{P}_3(x,y)$ (see Figs. 9a, 10a, and 11a) and the corresponding reference masks $\textbf{G}_\textrm{X}(x,y)$, $\textbf{G}_\textrm{Y}(x,y)$, and $\textbf{G}_\textrm{Z}(x,y)$. These reference masks represent the true object geometry. The left panel of Fig. 12 shows the reference mask $\textbf{G}_\textrm{Z}(x,y)$ for object “Z” (see Fig. 7b). The $\textrm{NMAE}$ is defined as follows

11 $$\begin{aligned} \textrm{NMAE} = \frac{1}{N} \sum _{(x,y)\in \textrm{ROI}} \left| \textbf{P}(x,y) - \textbf{G}(x,y) \right| \end{aligned}$$

where *N* denotes the number of pixels within the ROI, $\textbf{P}(x,y)$ represents the probability map normalized to the interval [0, 1], and $\textbf{G}(x,y)$ denotes the corresponding reference mask. Equation 11 is evaluated separately for each probability map and its corresponding reference mask. The spatial distribution of the absolute difference $\left| \textbf{P}_1(x,y) - \textbf{G}_\textrm{Z}(x,y) \right|$ within the ROI is visualized in the middle panel of Fig. 12 for object “Z”, corresponding to the probability map $\textbf{P}_1(x,y)$ shown in Fig. 11a.

To explicitly evaluate the geometric consistency of the reconstruction, $\textrm{IoU}_{\textrm{skel}}$ was employed as a complementary measure. Unlike boundary-based metrics, $\textrm{IoU}_{\textrm{skel}}$ assesses the alignment of the medial axes of the reconstructed object with the reference geometry and is therefore less sensitive to spatial broadening. Both the probability maps and the reference masks are reduced to one-pixel-wide skeletons, as illustrated for object “Z” in the right-hand panel of Fig. 12. The skeleton of the reference mask (white dotted line) represents the medial axis of the true object geometry. The skeleton of the probability map $\textbf{P}_1(x,y)$ (see Fig. 11a) (yellow dotted line) provides a compact representation of the spatially most stable region of the probability distribution and approximates the central geometric axis of the reconstructed object. This representation emphasizes the geometric structure rather than the extent of the object. The $\textrm{IoU}_{\textrm{skel}}$ is defined as

12 $$\begin{aligned} \textrm{IoU}_{\textrm{skel}} = \frac{\left| \mathcal {S}_{\textbf{P}} \cap \mathcal {S}_{\textbf{G}} \right| }{\left| \mathcal {S}_{\textbf{P}} \cup \mathcal {S}_{\textbf{G}} \right| } \end{aligned}$$

where $\mathcal {S}_{\textbf{P}}$ and $\mathcal {S}_{\textbf{G}}$ denote the skeletons of the probability map and the corresponding reference mask, respectively. Prior to evaluation, both skeletons were dilated by three pixels to allow small spatial misalignments between the reference and reconstructed skeletons.

**Fig. 13:**
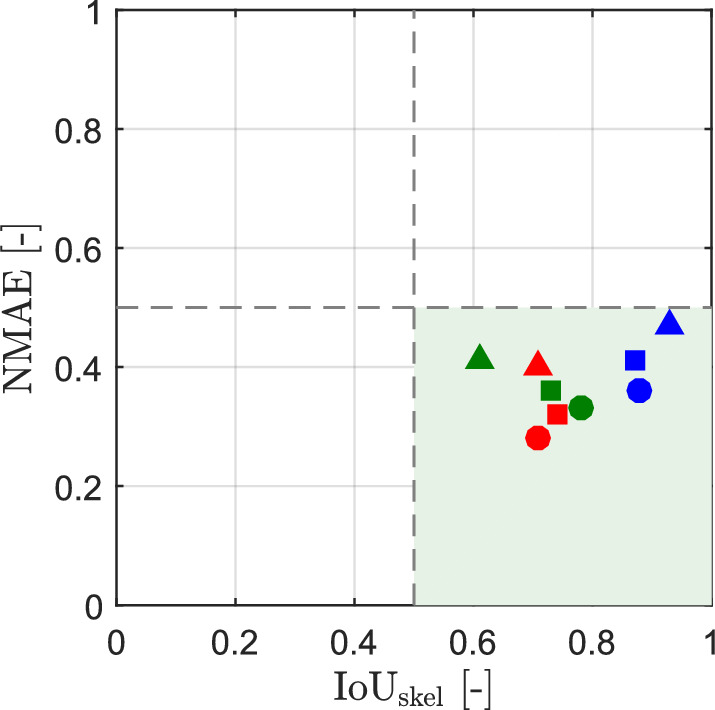
Scatter plot showing the relationship between NMAE and IoU$_{\textrm{skel}}$. Blue markers correspond to the X-shaped object, red markers to the Y-shaped object, and green markers to the Z-shaped object. Circular, square, and triangular markers indicate Gaussian filtering with standard deviations $\sigma _{\textrm{f1}}=2$, $\sigma _{\textrm{f2}}=4$, and $\sigma _{\textrm{f3}}=6$, respectively. IoU$_{\textrm{skel}}$ was computed using a dilation-based tolerance of 3 pixels to account for minor spatial misalignments. Dashed lines denote threshold values used to distinguish different reconstruction regimes, while the green shaded region indicates the parameter space where the combined values of NMAE and IoU$_{\textrm{skel}}$ correspond to acceptable reconstruction quality.

Figure 13 shows the joint NMAE–IoU$_{\textrm{skel}}$ evaluation of the results for all object geometries and filtering settings (see Figs. 9a, 10a, and 11a). The joint interpretation of NMAE and IoU$_{\textrm{skel}}$ enables a compact classification of the characteristics of the reconstruction. Low NMAE with high IoU$_{\textrm{skel}}$ indicates correct probability values and geometry. Low NMAE with low IoU$_{\textrm{skel}}$ indicates agreement in spatially averaged values but geometric distortions, such as missing branches or incorrect connectivity. High NMAE and low IoU$_{\textrm{skel}}$ indicate both amplitude errors and incorrect geometry. High IoU$_{\textrm{skel}}$ with elevated NMAE indicates correct localization of the object axis, but systematic deviations in object extent, such as over- or underestimation of object thickness.

### Extended results and analysis

This section provides additional experimental insight into the practical behavior of the proposed method beyond the baseline measurements. In particular, it examines how the measured effective conductivity $\sigma _{\textrm{e}}$ responds to controlled variations in the depth of the object and the lateral position beneath the loop pair. These outcomes are intended to characterize fundamental trends and limitations of the current configuration while maintaining identical measurement, excitation, and inversion settings.

#### Burial depth

In order to assess the achievable detection depth of the proposed method, an additional burial-depth experiment was conducted. The primary objective of this experiment is the reconstruction of the object at greater burial depth than in the baseline configuration. In addition, the effective conductivity $\sigma _{\textrm{e}}$ is analyzed as a function of burial depth for a selected antenna-pair configuration of the antenna array.

For the sake of clarity and reproducibility, all measurement parameters were kept identical to those described in section Measurement setup and Excitation pulse. This includes the pulse shape, pulse width $t_\textrm{w} = 125\,\textrm{ns}$, pulse amplitude $i_\textrm{m} = 0.5\,\textrm{A}$, and data acquisition settings. Owing to this strictly controlled setup, the influence of burial depth is effectively isolated.

First, the metallic object “X” (see Fig. 7b) was fully buried beneath progressively increasing layers of silica sand, with burial depths ranging from 1 mm to 30 mm, while all other experimental conditions remained unchanged. For each burial depth, the effective conductivity $\sigma _{\textrm{e}}$ was evaluated using the same analytical model and inversion procedure as the results described in section Measured Data and Analysis. The evaluated response $V_2^{\text {G}}(t)$ corresponds to the configuration between the transmitting antenna and the receiving loop $n = 2$ (see Fig. 4 and Fig. 8b), which forms part of the antenna array oriented at $45^\circ$, with a center-to-center spacing of $r_0 = 7.5\,\textrm{cm}$.

**Fig. 14:**
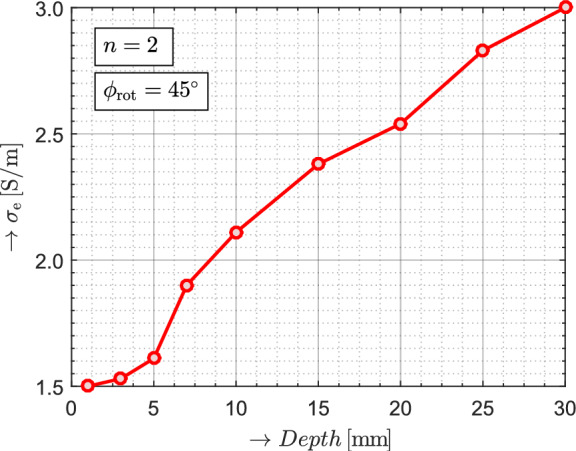
Dependence of the measured effective conductivity $\sigma _{\textrm{e}}$ on burial depth for object “X”, obtained for the antenna array orientation of $\phi _{\textrm{rot}} = 45^\circ$ using the transmitting loop and receiving loop $n = 2$, demonstrating a systematic reduction of conductivity contrast and convergence toward the reference conductivity $\sigma _{\textrm{ref}}$.

With increasing burial depth, the magnitude of the measured conductivity contrast systematically decreases, indicating a gradual convergence of the measured $\sigma _{\textrm{e}}$ towards the reference conductivity $\sigma _{\textrm{ref}}$. This trend is consistently observed throughout the investigated depth range, as shown in Fig. 14, and reflects the combined effects of EM field attenuation and spatial averaging inherent to the measurement geometry.

**Fig. 15:**
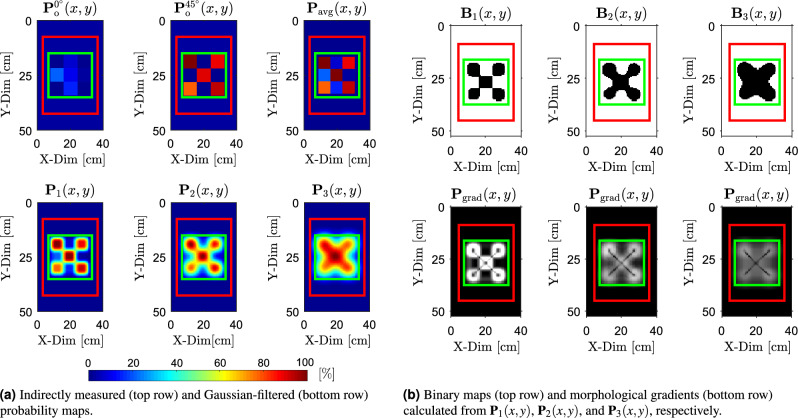
Results of detection for object “X” buried beneath a 20 mm layer of silica sand. (**a**) Raw and filtered probability maps, while (**b**) presents the corresponding binary segmentations and morphological gradients calculated from the probability maps.

A burial depth of 20 mm was selected for full reconstruction of object “X”, since at this depth the transient voltage responses show only weak variations while still maintaining a measurable contrast relative to the background, as also demonstrated in Fig. 14. The matrices containing absolute, non-normalized values of $\sigma _{\textrm{e}}$ for both measurement orientations $\phi _{\textrm{rot}} = 0^\circ$ and $\phi _{\textrm{rot}} = 45^\circ$ are shown below. The corresponding normalized probability maps are presented in Fig. 15. All reconstruction parameters, including binarization thresholds and morphological operations, were maintained constant throughout the signal processing, as in the results shown in Figs. 9, 10, and 11.

$$\begin{aligned} & \begin{array}{@{\hspace{2cm}} c@{\hspace{2cm}}c} \boldsymbol{\sigma }_{\text {e}, \text {X}}^{0^\circ }(x, y) = \begin{bmatrix} 3.02 & 2.96 & 3.02 \\ 2.89 & 2.95 & 3.01 \\ 3.02 & 2.92 & 3.02 \end{bmatrix} & \boldsymbol{\sigma }_{\text {e}, \text {X}}^{45^\circ }(x, y) = \begin{bmatrix} 3.02 & 2.46 & 3.02 \\ 2.58 & 2.52 & 2.52 \\ 3.02 & 2.54 & 3.02 \end{bmatrix} \end{array} \end{aligned}$$

#### Lateral offset

This section examines the sensitivity of the proposed method to small lateral displacements of a conductive target. The analysis focuses on the influence of positional offsets on the measured effective conductivity. The excitation conditions were kept constant as in section Measurement setup and Excitation pulse. This includes the pulse shape, pulse width $t_\textrm{w} = 125~\textrm{ns}$, and current amplitude $i_\textrm{m} = 0.5~\textrm{A}$. The lateral-offset (LO) experiment was conducted using a single transmit–receive loop pair with a spacing of $r_0 = 7.5~\textrm{cm}$, identical to the spacing used in the main experimental configuration.

**Fig. 16:**
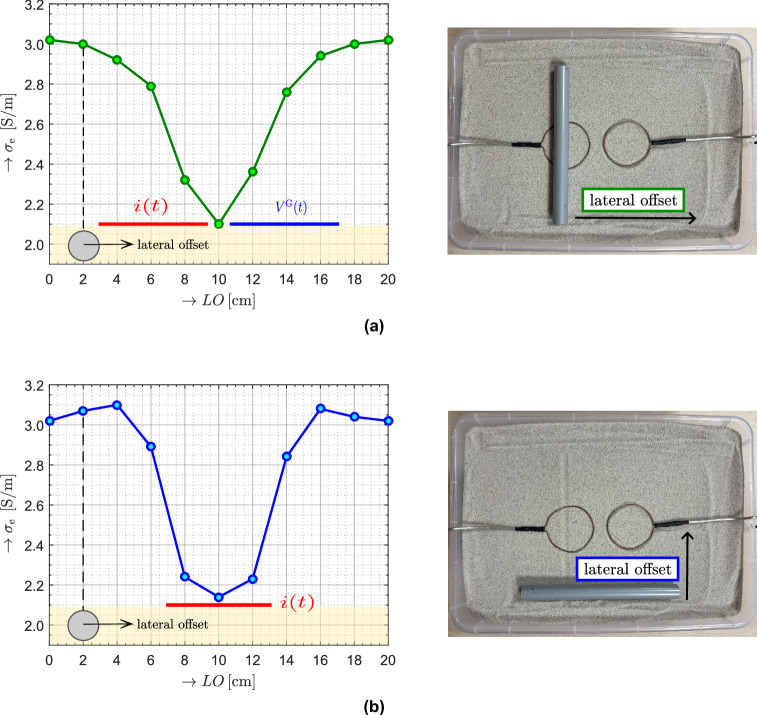
Lateral-offset sensitivity of the measured $\sigma _{\textrm{e}}$ for a metallic object fully buried beneath a $10~ \textrm{mm}$ layer of silica sand. (**a**) Displacement parallel to the line connecting the loop centers. (**b**) Displacement perpendicular to the line connecting the loop centers. The photographs illustrate the corresponding real measurement configurations, where the object is shown placed on the surface for clarity, while in the actual experiment it was fully buried beneath the sand layer.

The object consisted of a hollow metallic tube with a circular cross section, a diameter of $\approx 20~\textrm{mm}$, and a length of $20~\textrm{cm}$, fully buried beneath a $10~\textrm{mm}$ layer of silica sand. The object was laterally displaced in discrete steps of $2 ~\textrm{cm}$, referenced to the tube axis. Displacements parallel to the line connecting the loop centers are shown in Fig. 16a. Displacements perpendicular to this line, between the two loops, are shown in Fig. 16b. A burial depth of 10 mm was selected as an independent reference depth. At this depth, the lateral-offset response for a centered object shows close agreement with the corresponding point of the burial-depth analysis (see Fig. 14), despite minor differences in target geometry. The depictions in Fig. 16a and b are illustrative only and show the object placed on the surface for clarity.

The measured curves indicate that the evaluated effective conductivity $\sigma _{\textrm{e}}$ depends strongly on the lateral position of the object relative to the loop pair. The largest deviation from the background value occurs when the object is located near the midpoint between the two loops. As the object is displaced away from this position, the response weakens and $\sigma _{\textrm{e}}$ smoothly approaches the reference level $\sigma _{\textrm{ref}}$. Both displacement directions exhibit a similar overall trend, characterized by a distinct minimum and a symmetric return toward the background. Minor differences between the two curves reflect the directional sensitivity imposed by the loop geometry.

## Conclusion

In this paper, we present a TDEM approach to the reliable imaging of buried targets using only two rapid measurements via a simple and cost-effective antenna system. Additionally, this method builds upon a low-complexity and easy-to-implement processing algorithm^18^ that has been extended within this study to suit radar-based imaging of buried conductive targets. This algorithm was applied to real measured data from the antenna array, yielding accurate shapes of buried metallic objects. Thus, the reliability and functionality of the algorithm were thoroughly validated.

During the experiment, three different configurations of metallic targets were employed to demonstrate the ability of the method to detect objects with varying geometric complexity. The results demonstrate that the method can accurately reconstruct the shape of buried objects from only two antenna array positions. Despite these minimal measurement and computational requirements, the reconstructed profiles closely match the actual geometries. This confirms the effectiveness of the proposed approach for low-cost, real-time subsurface imaging.

The proposed method holds strong promise for practical use in many areas as near-surface geophysical applications, structural health monitoring, and UXO detection. Future development will aim at enhancing the resolution and extending the applicability through multi-position measurements and real-time implementations.

## Data Availability

The code scripts that support the findings of this study are publicly available at https://doi.org/10.5281/zenodo.18939967.

## References

[1] Griffiths, D. H. & King, R. F. Applied Geophysics for Geologists and Engineers: The Elements of Geophysical Prospecting (Elsevier, 2013).

[2] Yin, Y. et al. Integrated geophysical prospecting for deep ore detection in the Yongxin gold mining area, Heilongjiang, China. *Sci. Rep.* **15**, 7258. 10.1038/s41598-025-92108-3 (2025). 40025120 10.1038/s41598-025-92108-3 PMC11873187

[3] Daniels, D. J. A review of GPR for landmine detection. *Sens. Imaging* **7**, 90–123. 10.1007/s11220-006-0024-5 (2006).

[4] Montoya, T. P. & Smith, G. S. Land mine detection using a ground-penetrating radar based on resistively loaded vee dipoles. *IEEE Trans. Antennas Propag.* **47**, 1795–1806. 10.1109/8.817655 (1999).

[5] Gupta, M., Khan, M. A., Butola, R. & Singari, R. M. Advances in applications of non-destructive testing (NDT): A review. *Advances in Materials and Processing Technologies* **8**, 2286–2307. 10.1080/2374068X.2021.1909332 (2021).

[6] Batrakov, D., Batrakova, A. & Antyufeyeva, M. Combined GPR data analysis technique for diagnostics of structures with thin near-surface layers. *Diagnostyka* **19**, 11–20. 10.29354/diag/91489 (2018).

[7] Li, X., Davis, S. K., Hagness, S. C., van der Weide, D. W. & Veen, B. D. V. Microwave imaging via space-time beamforming: Experimental investigation of tumor detection in multilayer breast phantoms. *IEEE Trans. Microw. Theory Tech.* **52**, 1856–1865. 10.1109/TMTT.2004.832686 (2004).

[8] Lalitha, K. & Manjula, J. Non-invasive microwave head imaging to detect tumors and to estimate their size and location. *Phys. Med.* **13**, 100047. 10.1016/j.phmed.2022.100047 (2022).

[9] Baker, G., Jordan, T. & Pardy, J. *An introduction to ground penetrating radar (GPR)* **432**, 1–18 (2007).

[10] Liu, C., Han, P. & Kang, J. Integrating short-time linear canonical transform and joint space-time-frequency analysis for advanced representation of subsurface information in ground penetrating radar. *Sci. Rep.* **15**, 23315. 10.1038/s41598-025-06795-z (2025). 40604081 10.1038/s41598-025-06795-z PMC12223193

[11] Meng, B. et al. Study on the effect of pulsed eddy current lift-off characteristics for feeding metallic foreign objects detection in coal mine crushers. *Sci. Rep.* **15**, 23775. 10.1038/s41598-025-08185-x (2025). 40610503 10.1038/s41598-025-08185-x PMC12229576

[12] Trung, L. et al. Flexible eddy current array measurement system for crack detection in weld zones of steel structures. *Commun. Eng.* **4**, 132. 10.1038/s44172-025-00472-9 (2025). 40715355 10.1038/s44172-025-00472-9 PMC12297357

[13] Nabighian, M. N. & Macnae, J. C. Time domain electromagnetic prospecting methods. In Nabighian, M. N. (ed.) Electromagnetic Methods in Applied Geophysics, vol. 1 of Investigations in Geophysics No. 3, 427–514, https://doi.org/10.1190/1.9781560802686.ch6 (Society of Exploration Geophysicists, Tulsa, Oklahoma, 1991).

[14] Chen, C.-C. & Peters, L. Buried unexploded ordnance identification via complex natural resonances. *IEEE Trans. Antennas Propag.* **45**, 1645–1654. 10.1109/8.650076 (1997).

[15] Yang, Y. et al. Geological exploration of coal mine burnt rock and waterlogged area boundary based on transient electromagnetic and high-density electrical resistivity. *Sci. Rep.* **14**, 5105. 10.1038/s41598-024-55496-6 (2024). 38429304 10.1038/s41598-024-55496-6 PMC10907688

[16] Cao, B. et al. Research on comprehensive detection and visualize of hidden cavity goaf. *Sci. Rep.* **12**, 22309. 10.1038/s41598-022-26680-3 (2022). 36566311 10.1038/s41598-022-26680-3 PMC9789993

[17] Baum, C. E. Detection and Identification of Visually Obscured Targets (Routledge, 1998), 1st edn.

[18] Doležal, T., Štumpf, M., Qirui, H. & Franek, O. Measurement of electrical conductivity of human tissue: A feasibility study of a novel time-domain approach. *Measurement*10.1016/j.measurement.2024.115763 (2025).

[19] Štumpf, M., Antonini, G., Lager, I. E. & Vandenbosch, G. A. E. Time-domain electromagnetic-field transmission between small-loop antennas on a half-space with conductive and dielectric properties. *IEEE Trans. Antennas Propag.* **68**, 938–946. 10.1109/TAP.2019.2943323 (2020).

[20] Štumpf, M. & Antonini, G. Transient close-range electromagnetic field coupling between loop antennas at the interface of dissipative half-spaces. *IEEE Trans. Antennas Propag.* **69**, 1805–1808. 10.1109/TAP.2020.3016866 (2021).

